# Mechanisms and Signaling Associated with LPDBD Plasma Mediated Growth Improvement in Wheat

**DOI:** 10.1038/s41598-018-28960-3

**Published:** 2018-07-12

**Authors:** Md. Mosiur Rahman, Salek Ahmed Sajib, Md. Sifat Rahi, Sharaban Tahura, Nepal Chandra Roy, Sarwar Parvez, Md. Abu Reza, Mamunur Rashid Talukder, Ahmad Humayan Kabir

**Affiliations:** 1 Molecular Plant Physiology Laboratory, Department of Botany, University of Rajshahi, Rajshahi, 6205 Bangladesh; 2 Molecular Biology and Protein Science Laboratory, Department of Genetic Engineering and Biotechnology, University of Rajshahi, Rajshahi, 6205 Bangladesh; 3 Plasma Science and Technology Laboratory, Department of Applied Physics and Electronic Engineering, University of Rajshahi, Rajshahi, 6205 Bangladesh

## Abstract

This study investigates the effect and mechanisms of low pressure dielectric barrier discharge (LPDBD) produced with Ar/O_2_ and Ar/Air technique causing biological stimulation leading to improved germination and growth in wheat. Both plasma treatments caused rougher and chapped seed surface along with noticeable improvement in seed germination in wheat. Beside this, seed H_2_O_2_ concentration significantly increased compared to controls subjected to Ar/O_2_ and Ar/Air while this phenomenon was more pronounced due to Ar/Air plasma. Analysis of plants grown from the plasma treated seeds showed significant improvement in shoot characteristics, iron concentration, total soluble protein and sugar concentration in comparison with the controls more efficiently due to Ar/O_2_ plasma than that of Ar/Air. Further, none of the plasma treatments caused membrane damage or cell death in root and shoot of wheat. Interestingly, Ar/O_2_ treated plants showed a significant increase (2-fold) of H_2_O_2_ compared to controls in both root and shoot, while Ar/Air plasma caused no changes in H_2_O_2_. This phenomenon was supported by the biochemical and molecular evidence of SOD, APX and CAT in wheat plants. Plants derived from Ar/O_2_ treated seeds demonstrated a significant increase in SOD activity and *TaSOD* expression in roots of wheat, while APX and CAT activities along with *TaCAT and TaAPX* expression showed no significant changes. In contrast, Ar/Air plasma caused a significant increase only in APX activity in the shoot. This suggests that Ar/O_2_ plasma caused a slight induction in H_2_O_2_ accumulation without triggering the H_2_O_2_ scavengers (APX and CAT) and thus, efficiency affect growth and development in wheat plants. Further, grafting of control and Ar/O_2_ treated plants showed a significant increase in shoot biomass and H_2_O_2_ concentration in grafts having Ar/O_2_ rootstock regardless of the type scion attached to it. It indicates that signal driving Ar/O_2_ plasma mediated growth improvement in wheat is possibly originated in roots. Taken together, this paper delivers new insight into the mechanistic basis for growth improvement by LPDBD technique.

## Introduction

The production of crops is under threat in many countries of the world for overpopulation and reduced agricultural lands caused by rapid urbanization and industrialization. Although wheat (*Triticum aestivum* L.) is one of major stables crops in many countries, the production of wheat is decreasing due to several climatic (temperature, humidity, and drought, etc) and manmade factors^1–3^. Furthermore, intensive use of chemical fertilizers and pesticides used for increased yield brings hazards to human health and environment. Therefore, strategy and technology having no environmental risks are currently being focused on agriculturists.

Very recently, plasma technology is proven to have agronomic importance in few crops. Improvement in seed germination and growth have been demonstrated in few plant species^4–7^ by atmospheric pressure cold plasma (APCP). In addition, cold plasma treatment has been reported to modify seed wettability and germination in plants^8^. Due to its chemical-free capacity to induce germination and growth, plasma treatment is drawing much attention. In addition, atmospheric pressure cold plasmas APCPs are also found to be useful to inhibit seed contamination^9,10^, water uptake mechanisms^11^, and drought tolerance^3^. Most of the reports are concerned with atmospheric pressure dielectric barrier discharge (APDBD) plasmas. However, low pressure dielectric barrier discharge (LPDBD) technique is a vital source of non-thermal plasma due to its stability and large volume as compared to atmospheric pressure plasma source. Its potential effects are also applicable to the environment, energy conversion, biology, and sterilization^12–14^.

Seed germination and subsequent growth and development of plants lie on several physiological and biochemical indicators. Hydrogen peroxide (H_2_O_2_) is a reactive molecule that plays a dual role in plant physiological and developmental processes along with stress tolerance. The positive and negative roles of H_2_O_2_ in plant systems depend on its physiological conditions, concentrations and process specificities^15^. Although the plasma treatment achieved enhancement of seed germination and plant growth, it is considered to be challenging to optimize the species in the discharge that might produce elevated reactive oxygen species (ROS), such as superoxide anion (O_2_∙-) and hydroxyl radical (∙OH) and especially H_2_O_2_. While ROS and H_2_O_2_ function during imbibition and germination^16^, excess of these molecules cause oxidative damage leading cell death, membrane damage and protein degradation in plants. H_2_O_2_ is also considered as a signaling hub for the regulation of seed dormancy, germination, and antioxidant defense^15^. Another bioactive signaling molecule, nitric oxide (NO) participates in the control and regulation in many stages of plant development^17,18^. In addition, the role of NO in the breaking of seed dormancy and germination has also been reported^19,20^.

It was evident that LFGD plasma stimulates seed germination and growth in wheat^21^. However, the mechanisms associated with this exciting fact was not yet investigated in any plant species. Therefore, we combined a series of physiological, biochemical and molecular investigations to elucidate it in wheat. We also performed reciprocal grafting and signaling analysis to find out the origin of signaling that wheat posses due to plasma treatment.

## Results

### Changes in seed texture

**Figure 1:**
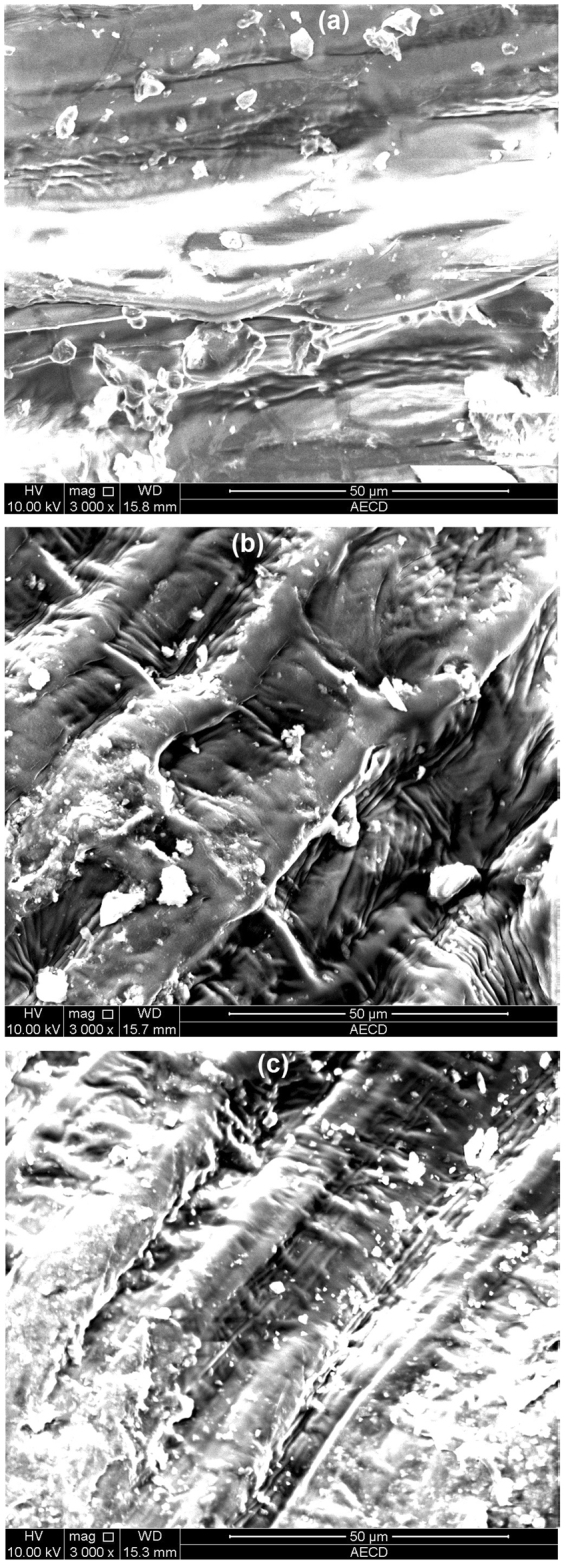
SEM images of the surfaces of wheat seeds treated for 90 s with (**a**) control (no plasma), (**b**) Ar/O_2_ and (**c**) Ar/Air. Scale bar is 50 *μm*.

The wheat seeds are treated by the three types of LPDBD Ar/O_2_ and Ar/Air (Fig. 1). The surface of wheat grains was much rougher as compared to that of control seed surface. Further, the seed coat becomes eroded and chapped by plasma treatment (Fig. 1).

### Germination, hydrogen peroxide (H_2_O_2_) and nitric oxide (NO) analysis

**Figure 2:**
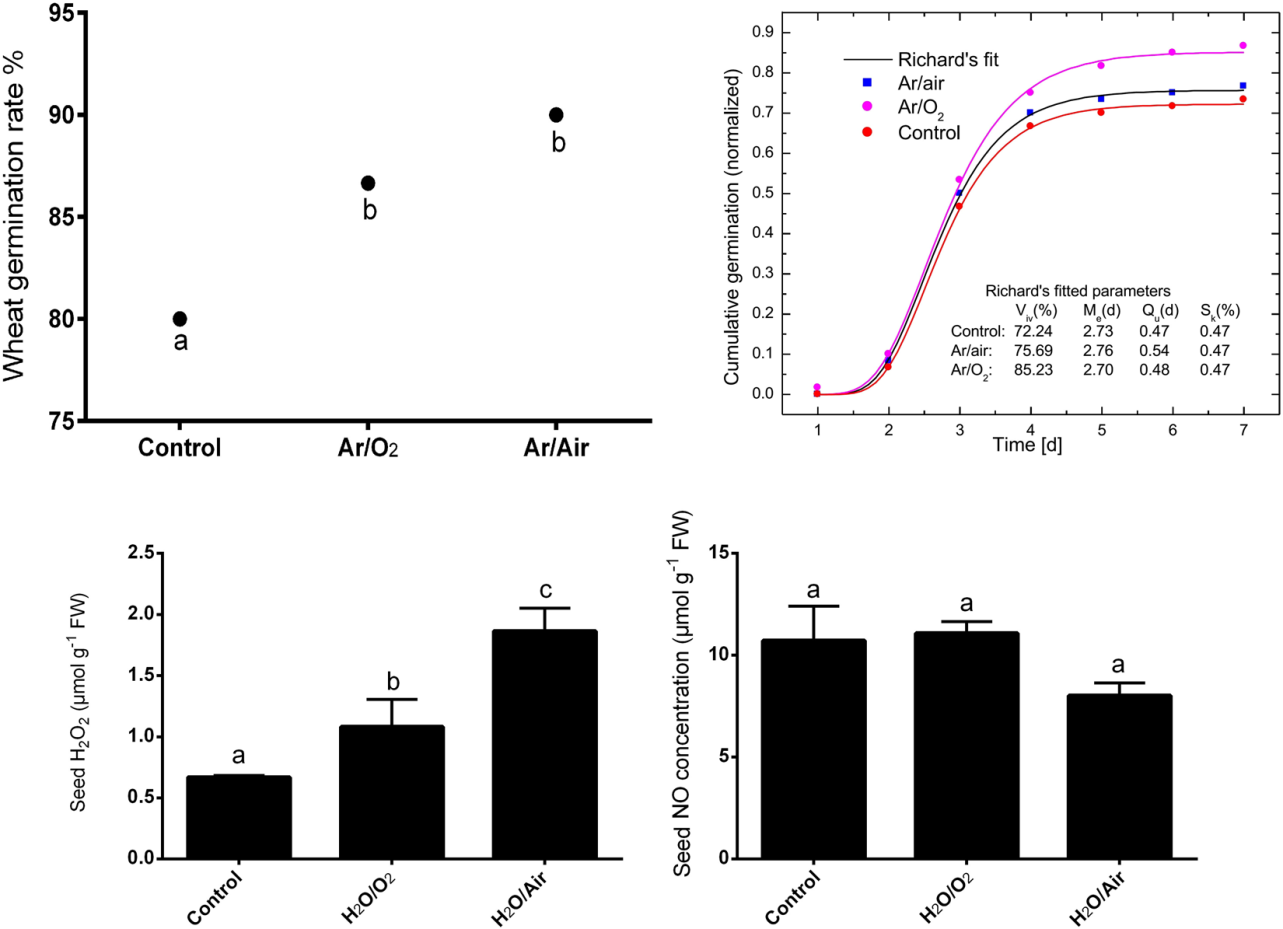
Germination rate, Richard’s fit*, H_2_O_2,_ and NO concentrations in seeds of wheat treated with Ar/O_2_ and Ar/Air plasmas. Different letters indicate significant differences between mean ± SD of treatments (n = 3) at a P < 0.05 significance level. *Richard’s fit (The population parameters, obtained from the fitting of cumulative germination of control and treated wheat seeds by Richard’s function, are: *V*_*iv*_− index of viability, *M*_*e*_− median germination time (indicates germination speed), *Q*_*u*_− dispersion (uniformity of seedling growth), *S*_*k*_− skewness. The values of the parameters are mentioned in the figure.

Both plasma treatments (Ar/O_2_ and Ar/Air) improved germination rate and seedling vigor in wheat seeds compared to the seeds germinated without plasma treatment although Ar/Air was more efficient than Ar/O_2_ (Fig. 2). Moreover, the germination data were well fitted by the Richard’s function as shown in Fig. 2. Further, H_2_O_2_ concentration significantly increased in seeds of wheat plants treated by Ar/O_2_ and Ar/Air plasma in comparison with non-treated controls while the increase was more pronounced due to Ar/Air than Ar/O_2_ (Fig. 2). However, any of the plasma treatment caused significant changes in NO concentration in wheat seeds (Fig. 2).

**Figure 3:**
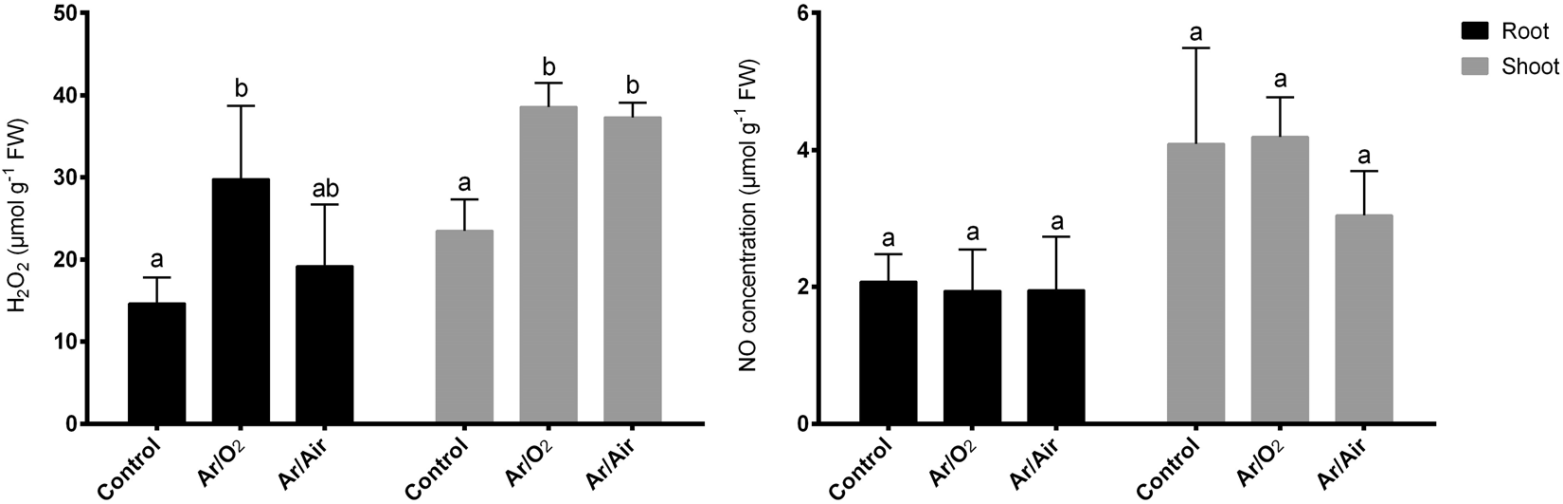
H_2_O_2,_ and NO concentrations in wheat plants grown from the seeds treated with Ar/O_2_ and Ar/Air plasmas. Different letters indicate significant differences between mean ± SD of treatments (n = 3) at a P < 0.05 significance level.

We further determined the concentration of H_2_O_2_ and NO in root and shoot of wheat plants germinated from seeds treated with plasma. We found that H_2_O_2_ concentration significantly increased in comparison with controls in both root and shoot when the plants were treated with Ar/O_2_ (Fig. 3). However, Ar/Air treatment showed no significant changes in H_2_O_2_ concentration in either root or shoot compared with controls (Fig. 3). In addition, no significant changes in NO concentration were observed in either root or shoot of wheat plants grown from the seeds treated with Ar/O_2_ and Ar/Air (Fig. 3).

### Morpho-physiological characteristics of wheat seedlings

Any of the plasma treatment (Ar/O_2_ and H_2_O/Air) caused significant changes in root length and root dry weight in wheat seedlings compared to controls. Moreover, seedlings showed a significant decrease in root length while seeds were treated with Ar/Air compared to non-treated controls (Table 1). However, shoot height and shoot dry weight significantly increased in seedlings treated with H_2_O/O_2_, while there shoot features showed no changes in seedlings treated with Ar/Air compared with controls (Table 1). In addition, no changes in total chlorophyll concentrations (*a* and *b*) were observed in leaves of wheat plants germinated from seeds treated with any of the plasma compared to controls (Table 1).

**Table 1:** Morpho-physiological characteristics of wheat plants grown from the seeds treated with Ar/O_2_ and Ar/Air plasmas.

Features	Root length (cm)	Root DW (g)	Shoot length (cm)	Shoot DW (g)	Chlorophyll (*a* + *b*)
Control	7.3 ± 0.92^b^	0.058 ± 0.002^b^	12.9 ± 0.55^a^	0.079 ± 0.013^a^	80.2 ± 7.90^a^
Ar/O_2_	6.1 ± 0.35^ab^	0.050 ± 0.017^b^	16.5 ± 0.98^b^	0.154 ± 0.031^b^	82.5 ± 17.5^a^
Ar/Air	5.6 ± 0.46^a^	0.044 ± 0.004^b^	13.7 ± 1.35^ab^	0.098 ± 0.018^a^	75.7 ± 5.90^a^

Different letters indicate significant differences between mean ± SD of treatments (n = 3) at a P < 0.05 significance level.

### Changes in biochemical characteristics

**Figure 4:**
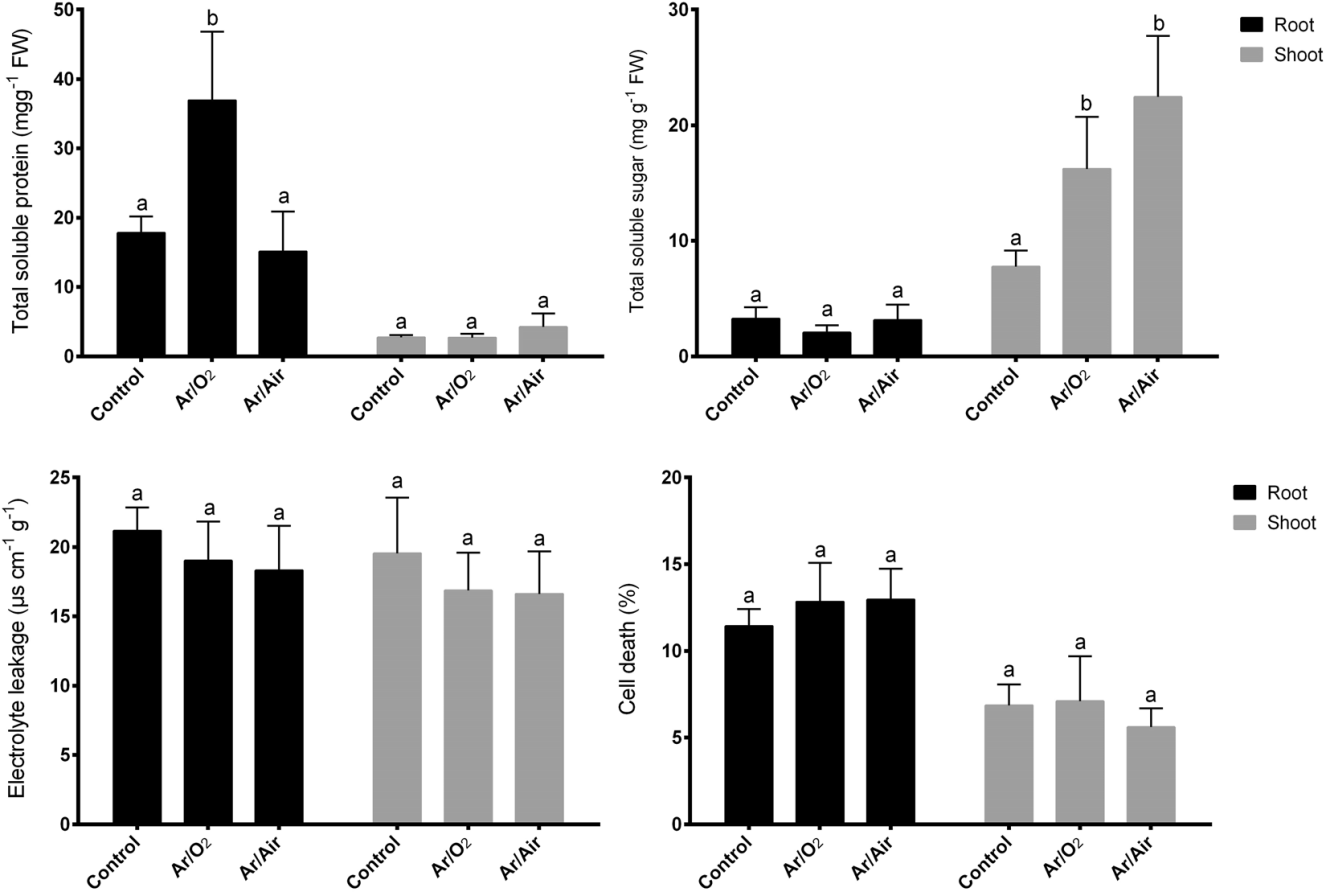
Total soluble protein, total soluble sugar, electrolyte leakage and cell death of plants grown from the seeds treated with Ar/O_2_ and Ar/Air plasmas. Different letters indicate significant differences between mean ± SD of treatments (n = 3) at a P < 0.05 significance level.

Wheat plants germinated from seeds treated with Ar/O_2_ and Ar/Air showed a significant increase and no differences in total soluble protein in roots compared with controls, respectively (Fig. 4). However, total soluble protein in shoot showed no significant changes in plants grown from the seeds treated with any of the plasmas (Fig. 4). Further, total soluble sugar did not show any changes in roots due to plasma treatment compared with controls (Fig. 4). However, both Ar/O_2_ and Ar/Air plasmas significantly increased total soluble sugar concentrations in shoot compared with the plants grown without plasma treatments (Fig. 4). In addition, no significant changes in electrolyte leakage and cell death (%) were observed in root and shoot of wheat plants germinated from seeds treated with either Ar/O_2_ or Ar/Air compared with controls (Fig. 4).

### Analysis of antioxidant enzymes (SOD, APX, CAT) in seeds and seedlings

**Figure 5:**
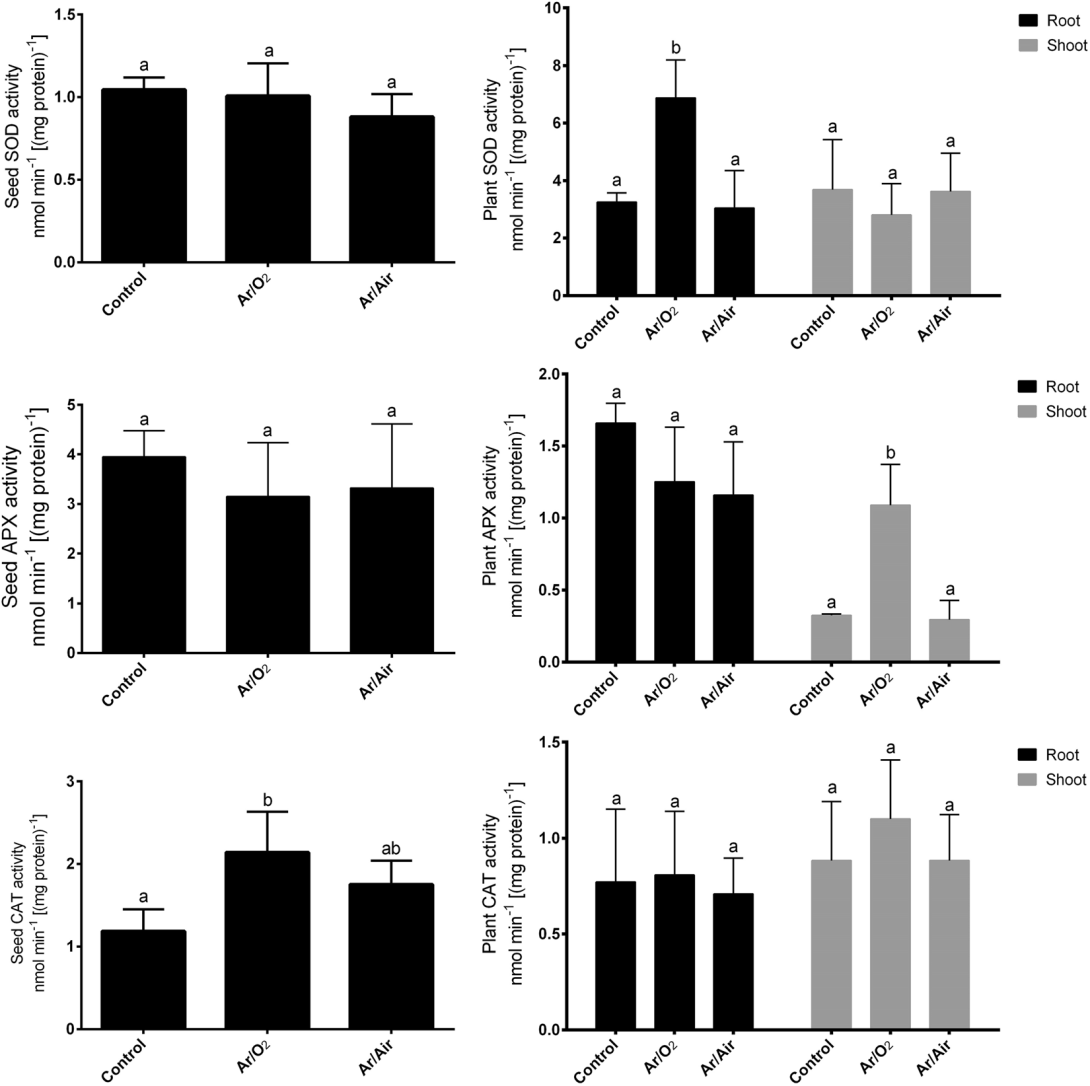
SOD, APX and CAT activities in seeds and plants grown from the seeds treated with Ar/O_2_ and Ar/Air plasmas. Different letters indicate significant differences between mean ± SD of treatments (n = 3) at a P < 0.05 significance level.

In seeds, SOD and APX activities showed no significant changes due to any of the plasma treatments compared with the non-treated seeds (Fig. 5). However, Ar/O_2_ treatment caused a significant increase in CAT activity in seeds compared with controls. Further, Ar/Air treatment showed similar CAT activity to that of control seeds (Fig. 5).

In plants, SOD activity significantly increased in roots of plants grown from the seeds treated with Ar/O_2_ compared with controls. However, no changes in SOD activity was monitored in the shoot of plants grown from the seeds treated with either Ar/O_2_ or Ar/Air plasma (Fig. 5). In addition, no changes in APX activity was observed in the root of plants grown from the seeds treated with either Ar/O_2_ or Ar/Air plasma compared with controls. However, APX activity significantly increased due to Ar/O_2_ treatment in the shoot of wheat plants compared with non-treated controls (Fig. 5). In this study, CAT activity did not show any changes in either root or shoot of wheat plants germinated from seeds treated with Ar/O_2_ or Ar/Air plasma compared to the controls (Fig. 5).

### Elemental analysis of Fe and Zn

**Figure 6:**
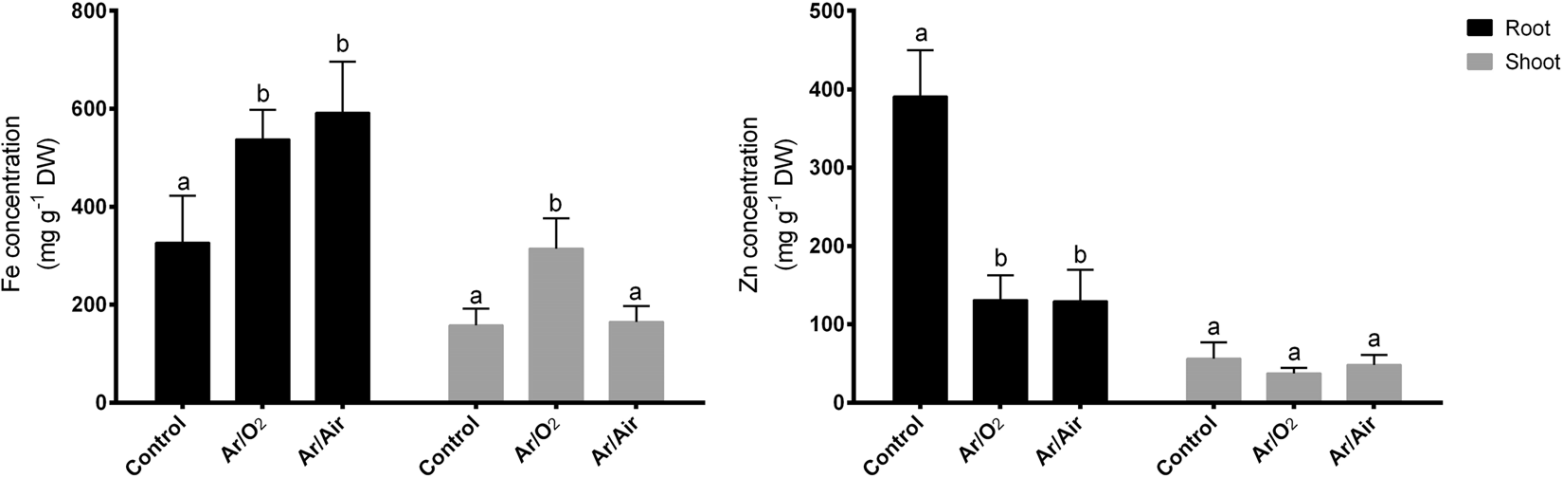
Fe and Zn concentrations in root and shoot of wheat plants grown from the seeds treated with Ar/O_2_ and Ar/Air plasmas. Different letters indicate significant differences between mean ± SD of treatments (n = 3) at a P < 0.05 significance level.

AAS analysis showed that Fe concentration significantly increased in both root and shoot of wheat plants originated from the seeds treated with Ar/O_2_ compared with non-treated controls (Fig. 6). However, Ar/Air plasma showed a significant increase of Fe only in roots compared with controls (Fig. 6). In addition, Zn concentration did not show any changes in the shoot but it significantly decreased in plants germinated from seeds treated with either Ar/O_2_ or Ar/Air (Fig. 6).

### Expression of genes related to antioxidant activities

**Figure 7:**
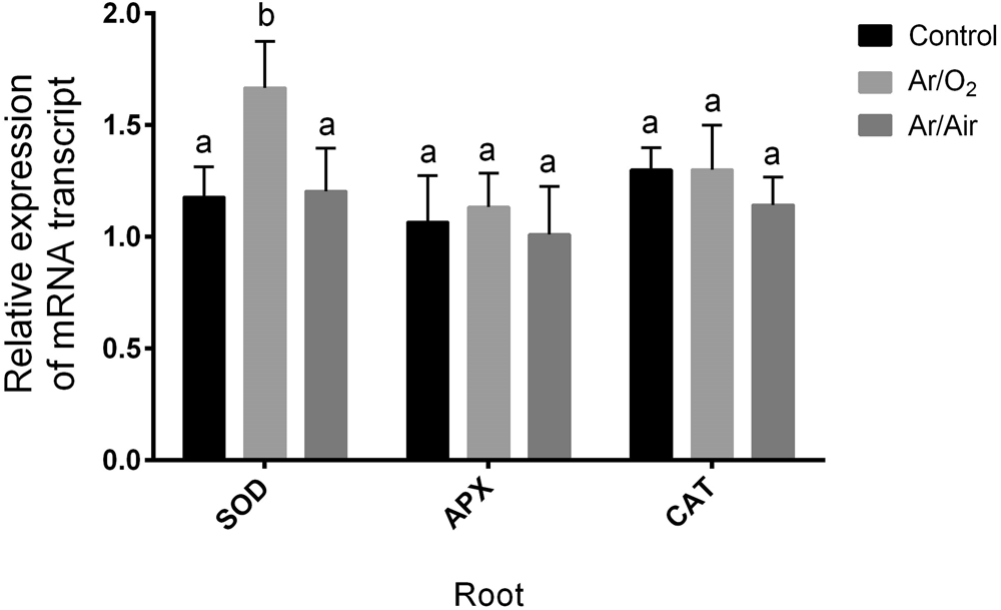
Quantitative analysis of expression pattern of *TaSOD*, *TaAPX* and *TaCAT* transcripts in roots of wheat plants grown from the seeds treated with Ar/O_2_ and Ar/Air plasmas. Different letters indicate significant differences between mean ± SD of treatments (n = 3) at a P < 0.05 significance level.

Our real-time PCR analysis showed no significant changes in *TaAPX* and *TaCAT* expression in roots of plants originated from seeds treated with either Ar/O_2_ or Ar/Air (Fig. 7). However, *TaSOD* expression significantly upregulated in roots of wheat plants treated at seed stage with Ar/O_2_ compared with non-treated controls (Fig. 7).

### Analysis of grafted plants

Based on our results, we have chosen Ar/O_2_ in grafting experiment as the most effective treatment. In this grafting study, root length and root dry weight showed no significant changes among the four types of grafts grown from the seeds treated with or without Ar/O_2_ plasma (Table 2). However, grafts having Ar/O_2_ plasma treated rootstock (type 2 and 4) attached to either control or Ar/O_2_ treated plants showed a significant increase in shoot height and shoot dry weight compared to the control self-graft (type 1) or control combined with Ar/O_2_ treated scion (type 3) grown from pre-treated seeds (Table 2).

**Table 2:** Morphological features and H_2_O_2_ concentration in different combinations of grafted plants grown from the seeds treated with Ar/O_2_ plasma.

Features	Root length (cm)	Root DW (g)	Shoot length (cm)	Shoot DW (g)	H_2_O_2_ (µmol g^−1^ FW)	
					root	shoot
Type 1	4.2 ± 0.75^a^	0.032 ± 0.003^a^	4.4 ± 0.49^a^	0.027 ± 0.003^a^	9.3 ± 2.75^a^	7.7 ± 3.74^a^
Type 2	4.3 ± 0.26^a^	0.032 ± 0.004^a^	5.6 ± 0.57^b^	0.039 ± 0.003^b^	18.4 ± 2.19^b^	18.0 ± 2.66^c^
Type 3	4.5 ± 0.75^a^	0.034 ± 0.004^a^	4.3 ± 0.55^a^	0.026 ± 0.007^a^	11.3 ± 0.60^a^	11.8 ± 1.58^ab^
Type 4	4.6 ± 0.40^a^	0.035 ± 0.005^a^	5.4 ± 0.25^b^	0.038 ± 0.003^b^	22.6 ± 4.58^b^	16.1 ± 2.33^bc^

Different letters in each column indicate significant differences between mean ± SD of treatments (n = 3) at a P < 0.05 significance level.

Type 1: control self grafting, type 2: H_2_O/O_2_ self grafting, type 3: control rootstock + H_2_O/O_2_ scion, Type 4: H_2_O/O_2_ rootstock + control scion.

## Discussion

Agronomic improvement in crop plants is highly desirable in order to fulfill the demand of the large population. Although plasma technology proved to be efficient in a very few plant species, the mechanistic basis for plasma-mediated improvement in germination and growth is still hazy. This comprehensive study reveals some new insights into the role of plasma technology to trigger the germination and growth of wheat plants.

First, LPDBD plasma treatment stimulated changes in seed surface morphology and functionalities. The changes in surface morphology and roughness may be associated with the enhanced water permeability into the seeds, and as a result, change in wetting behavior occurs. The seed surface contains irregular shape, different size and randomly distributed starch and protein granules^22,23^. In this study, the differences between the treated and untreated seeds in functionalities were also noticeable throughout the entire experiments. Also, LPDBD plasma treatments in our study produced different functional reactive species. The O_2_ containing functional groups are enriched the surface of the seeds that result in essential improvement of wettability and finally influenced the germination. It is reported that the incorporation of nitrogen on the seed surface enhances germination which is consistent with our findings^24^.

In addition, exposure of wheat seeds to the LPDBD plasma treatments (Ar/O_2_ and Ar/Air) showed some stimulating effects with respect to the germination rate and seedlings vigor. Previously, DBD (dielectric barrier discharge) plasma treatment reported being useful for enhancing seed germination rate, and vigor index in wheat plants^25,26^. In our study, the LPDBD Ar/Air plasma proved to be better than Ar/O_2_ to increase germination rate and thus, suggest that Ar/Air plasma could be more efficient while inducing seed germination is primarily desirable. Our extended study on the seeds further reveals the increase of H_2_O_2_ concentration in seeds treated with LPDBD plasmas, while the increase of H_2_O_2_ was more pronounced in Ar/Air plasma treated seeds. The correlation between increased seed germination rate and H_2_O_2_ concentration due to Ar/Air plasma suggest that induced H_2_O_2_ generated under this condition was efficient to trigger seed germination in wheat. H_2_O_2_ is recognized as a versatile molecule involved in germination and growth of plants. The accumulation of H_2_O_2_ is reported in seed physiology during imbibition, early germination stage and when seeds become hydrated^16,27,28^. However, another signaling molecule NO was not detected to be differentially changed due to any of the plasmas used in this study.

Further, H_2_O_2_ along with other ROS is often considered to be the main reason for seed deterioration and loss of seed vigor^29^. Our enzymatic analysis revealed the increased activity of CAT in seeds treated with Ar/O_2_ but not in Ar/Air plasma in comparison with non-treated seeds. This enzymatic data is correlated with the seed H_2_O_2_ concentration in the sense that CAT is one of the main antioxidant enzymes associated with the H_2_O_2_ scavenging. In other words, elevated CAT activity in seeds minimized the induction of H_2_O_2_ due to Ar/O_2_ plasma, while higher H_2_O_2_ level is detected as the CAT activity is not increased subjected to Ar/Air plasma in wheat seeds.

In most of the cases, Ar/O_2_ plasma was found to be more efficient that Ar/Air plasma for the morphological growth and development of wheat plants. In this study, we noticed that shoot height and dry matter accumulation significantly improved in plants grown hydroponically from the Ar/O_2_ plasma treated seeds. Similar agronomic improvements were previously reported in poppy and tomato plants subjected to atmospheric pressure cold plasmas^7,30^. Interestingly, our studies showed the increase of total soluble protein and sugar in either root or shoot due to LPDBD Ar/O_2_ plasma treatment. It does indicate the effectiveness of LPDBD Ar/O_2_ on enhanced cell metabolism leading to enhanced growth and development in wheat plants. Ar/O_2_ functions as feed gases that might increase nitrogen accumulation and enhance the activity of nitrate reductase as well as glutamine synthetase contributing towards increased seedling growth in wheat plants. This observation is consistent with the previous results in wheat and Suaeda plants^5,31^. Interestingly, we observed a significant increase of Fe in both root and shoot wheat plants subjected to Ar/O_2_ plasma treatment in seeds. Iron uptake is dependent on the plant’s ability to reduce Fe^3+^ to Fe^2+^ through the electrons at the cell surface^32^. It pinpoints that Ar/O_2_ plasma treatment may also modulate electron discharge which in turn facilitate iron uptake in wheat plants.

Considering the generation of different species and the possibility of excess ROS accumulation of subjected to LPDBD plasma treatments (Ar/O_2_ and Ar/Air), we evaluated a number of biochemical characteristics in wheat plants associated with cellular response and damage. Results showed that none of the plasma treatments resulted in membrane damage and cell death in wheat plants. This further confirms the effectiveness and safety of plasma treatment for growth improvement in wheat.

In several previous reports, a number of antioxidant enzymes were reported to be involved with plasma mediated improvement in plants. However, neither of the reports focused on the background scenario of antioxidant defense that plants induce in special conditions. Although we initially considered both H_2_O_2_ and NO as signaling molecules, only H_2_O_2_ concentration increased in both root and shoot up to 2-fold in wheat plants grown from the seeds treated with Ar/O_2_ plasma. Correspondingly, CAT and APX which are mainly active against elevated H_2_O_2_ showed no significant changes in either root or shoot subjected to the Ar/O_2_ plasma treatment. This biochemical evidence was further verified by the gene expression analysis of *TaCAT* and *TaAPX* showed no significant changes in the same conditions. It does indicate that the slight increase of Ar/O_2_ plasma-induced H_2_O_2_ is not only useful for improving wheat growth but also not threatening or toxic to plants that might trigger antioxidant enzymes, such as CAT or APX. Consistently, plants derived from Ar/Air treated seeds showed no significant increase of H_2_O_2_ in tissues. This might occur due to the increased activity of APX activity or plants were not efficient to carry over H_2_O_2_ signaling at mature stage due Ar/Air plasma. APX is mainly involved in the fine-tuning of H_2_O_2_ detoxification via its sulfhydryl group^33^. Interestingly, SOD activity and its corresponding *TaSOD* gene significantly increased in roots of plants derived from Ar/O_2_ plasma. SOD is the frontline defense in plants to counteract superoxide O_2_^−^. Therefore, it reveals that Ar/O_2_ plasma treated for 6 minutes simultaneously regulate reactive O_2_^−^ and H_2_O_2_ to the optimum level in wheat plants.

## Methods

### Plasma production and species identifications

One copper disk type electrode (diameter 9 *mm*, thickness 0.5 *mm*) was placed at the lower end of the test tube (diameter 12 *mm*, length 50 *mm*). Another copper disk electrode (diameter 8 *mm*, thickness 0.5 *mm*) was covered by a glass tube (thickness 1 *mm*) and was placed at the upper end of the discharge tube as shown in the figure. The electrode spacing between two electrode was maintained at 40 *mm*. A high voltage (5–10 *kV*, 3–8 *kHz*) bipolar sinusoidal power supply was fed to the electrodes for plasma generation. Wheat seeds to be treated were placed in the discharge tube between two electrodes. The inside pressure of the chamber was reduced by a vacuum pump (*FY*−1*C*) and inside pressure was maintained at (~10 *torr*). The flows of *Ar*,*O*_2_ and *Air* to the chamber were controlled by three different gas flow meter *Yamato*, *KIT* and 115 *P* respectively. The waveforms of the discharge voltage and current are recorded with voltage (*HVP*−08) and current (CP−07C) probes, respectively, in combination with a digital oscilloscope (GDS-1000B). The power absorbed by the plasma was determined $P={\int }_{0}^{T}v(t)i(t)$, where *v*(*t*) and *i*(*t*) are the discharge voltage and current, respectively, by integrating over the period *T*. The amount of power absorbed by the plasma was ~45 *W* for *Ar*/*Air* gas mixture measured at: applied voltage 5 *kV*, frequency 4.5 kHz electrode spacing 60 *mm*. For species identification, the emitted spectra produced in the plasma are recorded with spectrometers (USB2000 + XR1, slit size: 25 *μm*, grating: 800 *lines*/*mm*, optical resolution: 1.7 *nm*, wave length range: 200−1100 *nm* and high resolution dual-channel spectrometer AvaSpec-2018, slit: 10 *μm*, gratting: 2400 *lines*/*mm*, optical resolution: 0.07 *nm*, wavelength range: 200−500 *nm*). The ROS and RNS produced were Ar/O_2_ (*Ar*:60%,*O*_2_: 40%) and Ar/Air (*Ar*: 60%,*O*_2_: 40%) the plasmas at voltage: 5 *kV*, electrode spacing: 40 *mm*, pressure (~10 *torr*) (Supplementary Figure S1).

The major electronic transitions of nitrogen second positive system N_2_(C^3^Π_u_−B^3^Π_g_) in the range 294−380 *nm* and the first negative system ${{\rm{N}}}_{2}^{+}({{\rm{B}}}^{2}{{\rm{\Sigma }}}_{{\rm{u}}}^{+}-{{\rm{X}}}^{2}{{\rm{\Sigma }}}_{{\rm{g}}}^{+})$ in the range 391–405 *nm* were found as RNS from Ar/Air plasma. Atomic transitions of N^+^ were detected at wavelength 517.52 nm and 668.22 nm. Form the figure it is seen that the transitions of O radicals occured at 777.1 and 844.2 nm for Ar/O_2_ plasma. Reactive oxygen species (ROS) was also observed as ionic transition of O^+^ and O_2_^+^ was found. The band transition of NO(*A*^2^Σ^+^→*X*^2^Π) was found in Ar/O_2_ plasma shown in inset of figure (Supplementary Fig. S1). Due to the presence of Ar gas the transition of Ar lines were detected in the spectrum in the range 685–914 nm and transition of *H*_*β*_ and *H*_*α*_ line were found for both Ar/Air and Ar/O_2_ plasmas. Rotational (T_rot_) and vibrational (T_vib_) temperatures were determined by simulating the first negative system ${{\rm{N}}}_{2}^{+}({{\rm{B}}}^{2}{{\rm{\Sigma }}}_{{\rm{u}}}^{+}-{{\rm{X}}}^{2}{{\rm{\Sigma }}}_{{\rm{g}}}^{+})$ with the aid of LIFBASE [21] spectroscopic software. The analysed results revealed that T_rot_ ≈ 306 K, T_vib_ ≈ 2.42 kK for *air* and T_rot_ ≈ 323 K, T_vib_ ≈ 2.58 kK for *air*/O_2_ plasmas measured at 5 kV with dissipated power ~45 W. The wheat seeds were selected randomly both for treatment and control. The experimental setup of plasma technique and seed placement with Ar/O_2_ and Ar/Air plasmas is shown in Supplementary Figure S1.

### Seed treatment and plant cultivation

The wheat seeds (BARI Gom 22) were selected randomly both for treatment and control. The seeds were treated by LPDBD plasma with the following gas compositions: Ar/Air and Ar/O_2_. The temperature of the gas composition was ~304 *K* measured by a thermometer at 2 *mm* from the electrodes. The wheat seeds were placed inside a tubular glass container made of 12 mm inner diameter glass tube. The length of glass container was 50 mm long. This arrangement was preferred for optimum surface treatment of wheat seeds. The treatment times followed for the seeds were 90 s.

Seedlings were then transplanted to the hydroponic culture^34^ supplemented with the following nutrient elements (µM):KNO_3_ (16000), Ca(NO_3_)_2_.4H_2_O (6000), NH_4_H_2_PO_4_ (1000), MgSO_4_.7H_2_O (2000), KCl (50), H_3_BO_3_ (25), Fe-EDTA (25), MnSO_4_.4H_2_O (2), ZnSO_4_ (2), Na_2_MoO_4_.2H_2_O (0.5) and CuSO_4_.5H_2_O (0.5). Seedlings were cultivated in 2 L container under 10 h light and 14 h dark (550–560 µmol s-1 per µA) in a growth cabinet. The pH of the cultivation media was adjusted to 6.0. Plants were cultivated for 4 days once transferred to solution culture and concurrently harvested.

### Germination percentage and test

The seeds (around 25) were placed in each 90 mm Petri dish containing two layers moistened with wet filter papers at the bottom for germination. The seed containing Petri dishes were incubated at 25 °C in an incubator for 3d. Afterwards, additional distilled water was added daily to maintain sufficient moisture content for germination. The germination percentage is recorded after 3d. The Richard’s function Y(t) is calculated as previously described^20^.

### Morphological features and chlorophyll (a and b) determination

For chlorophyll analysis, young fresh leaves were weighted and ground with mortar and pestle in 90% methanol. The sample mixtures were subsequently centrifuged at 12000 rpm for 5 min and the cell debris was discarded. Finally, the absorbance of the clear supernatant was recorded 662 nm (chlorophyll *a*) and 646 nm (chlorophyll *b*) by spectrophotometer (UV-1650PC, Shimadzu. The absorbance of the supernatant was then monitored at 662 nm (chlorophyll *a*) and 646 nm (chlorophyll *b*) by spectrophotometer (UV-1650PC, Shimadzu). The total concentration of chlorophyll (*a* and *b*) was obtained following the calculation^35^.

### Analysis of antioxidant enzymes (SOD, APX, and CAT) in seeds and plants

SOD, APX and CAT enzymes were extracted as previously described with slight modifications^36^. Briefly, tissues were ground in phosphate buffer (100 mM, pH 7.0) with mortar and pestle. The homogenate was centrifuged for 10 min (8000 rpm) before separating the supernatant in centrifuge tubes. For SOD analysis, 100 µl tissue extract was mixed with 50 mM sodium carbonate/bicarbonate buffer (pH 9.8), 0.1 mM EDTA and 0.6 mM epinephrine^37^. The adrenochrome formation was then read at 475 nm after 4 min in a UV-Vis spectrophotometer. APX activity was tested in a reaction mixture containing 0.1 mM EDTA, 50 mM potassium phosphate buffer (pH 7.0), 0.5 mM ascorbic acid, 0.1 mM H_2_O_2_, and 0.1 ml extract. The activity of APX was finally calculated using extinction coefficient of 2.8 mM^−1^ cm^−1^ based on the absorbance at 290 nm^38^. Further, CAT was analyzed in a reaction mixture supplemented with 100 mM potassium phosphate buffer (pH 7.0), 6% H_2_O_2_ and 100 µl root extract. Once extract is added, the changes in optical density were monitoredat 240 nm (extinction coefficient of 0.036 mM^−1^ cm^−1^) in a UV spectrophotometer at 30 s intervals up to 1 min.

### Determination of H_2_O_2_

For H_2_O_2_ analysis, washed root and shoot was homogenized in 0.1% trichloroacetic acid (TCA using mortar and pestle^39^. The homogenates were centrifuged at 10 000 rpm for 15 min to get rid of the cell debris. The clear supernatant was then mixed with potassium iodide (1 M) and phosphate buffer (10 mM, pH 7.0) and kept for 1 h at dark. Lastly, the optical density of the extract mixture was taken at 390 nm by a spectrophotometer (UV-1650PC, Shimadzu).

### Measurement of electrolyte leakage

Electrolyte leakage of root and shoot membrane was determined using a digital electrical conductivity meter^40^. Firstly, detached root and shoot tissue was washed with deionized water and placed in a 20 ml vial filled with water. The vials were incubated for 2 hours at room temperature on a shaker. Finally, the electrical conductivity of the vial solution was recorded.

### Determination of cell death

Analysis of cell death in root and shoot was performed following Evans blue method with some modifications^41^. Initially, separated root and shoot were incubated at room temperature in 0.25% Evans blue solution for 15 min. After that, the solution was replaced with 1.0 mL of 80% ethyl alcohol for 10 min. The samples were then incubated in a water bath at 50 °C for 15 min. Further, the samples were centrifuged at 12,000 rpm for 10 min. Finally, the absorbance of the clear supernatant was measured at 600 nm, and the cell death was calculated on the basis of fresh weight.

### Analysis of total soluble sugar

Soluble sugar in root and shoot was analysed as previously described^42^. Briefly, fresh root and shoot were homogenized in hot (90 °C) aqueous ethanol (v/v 80%) and centrifuged at 12000 rpm for 5 min. Subsequently, the clear supernatant was mixed with 0.2% of anthrone reagent. The sample mixtures were incubated in a boiling water bath for 8 min before placing on ice. The absorbance of the ice-cold samples was finally recorded at 620 nm.

### Estimation of total soluble protein

The total soluble protein was tested in both roots and shoots using spectrophotometer^43^. Firstly, washed root and shoot was weighed and homogenized with ice-cold mortar and pestle in assay buffer supplemeted with 2 mM EDTA (ethylenediaminetetraacetic acid), 50 mM Tris-HCl, pH 7.5 and 0.04% (v/v) 2-mercaptoethanol. The homogenates were then centrifuged at 12000 rpm for 10 min at 25 °C, and the supernatant was moved to glass cuvette filled with 1 ml CBB (Coomassie Brilliant Blue). Afterwards, total soluble protein was determined following the absorance of unknown samples taken at 595 nm in a spectrophotometer using the standard curve of BSA (bovine serum albumin).

### Determination of Fe and Zn in root and shoot

Harvested root and shoot tissues were initially detached and washed in CaSO_4_ (1 mM) for 5 min. The tissue samples were washed with deionized water for several times before drying at 80 °C for 2 d in the oven. Afterwards, samples were digested with 5 ml HNO_3_ and 2 ml HClO_4_ in a glass beaker and heated in a microwave oven. Fe and Zn were then determined by Flame Atomic Absorption Spectroscopy (AAS) attached to an ASC-6100 autosampler air-acetylene atomization gas mixture system (Model No. AA-6800, Shimadzu). Also, a standard known solution of Fe and Zn were prepared from their corresponding stock solutions^44^.

### NO analysis in root and shoot

NO was determined in root and shoot of wheat plants based on the changes of hemoglobin absorbance consequently of its conversion from oxyhemoglobin (HbO_2_) to methemoglobin (metHb) in the presence of NO^45^. Briefly, harvested root or shoot samples were ground in 1 ml of cooled NO buffer containing 0.1 M sodium acetate, 1 M NaCl, and 1% (w/v) ascorbic acid (pH 6.0). The homogenates were then centrifuged at 10,000 rpm for 5 min at 4 °C, and the supernatants are transferred to a centrifuge tube. Afterwards, the HbO_2_ solution stock (5 mM) was added to the samples and incubated for 5 min at room temperature. The rate of HbO_2_ to metHb conversion was evaluated at 401 nm.

### RNA isolation and gene expression analysis

Expression analysis of *TaSOD, TaAPX*, and *TaCAT* transcripts was performed in roots by quantitative qRT-PCR (reverse transcription PCR). Briefly, roots (50–70 mg) were ground in liquid nitrogen with a mortar and pestle to a fine powder. Afterwards, the total RNA was extracted according to the protocol instructed by SV Total RNA Isolation System (Cat. no. Z3100, Promega Corporation, USA). The superiority of RNA samples was subsequently verified by denaturing gel electrophoresis. Once the RNA is quantified by UV-Vis Spectrophotometer (NanoDrop 2000), the first-strand cDNA was synthesized as instructed by GoScript™ Reverse Transcription System (Cat no. A5001, Promega Corporation, USA). After that, cDNA samples were treated with RNase to eliminate possible RNA contamination. The real-time PCR analysis was performed in Eco^TM^ real-time PCR system controlled by Eco Software v4.0.7.0 (Illumina, USA). Sequences of each gene-specific primer were presented in Supplementary Table S1. Expression analysis was normalized with *Actin* as an internal control (Eco Software v4.0.7.0). The real-time PCR program used was as follows: 3 min at 95 °C, 40 cycles of 30 s at 94 °C, 15 s at 56 °C and 30 s at 72 °C.

### Grafting of control and Ar/O_2_ treated seedlings

Reciprocal grafting between control and Ar/O_2_ treated plants were performed on post-germinated young seedlings^44^. Briefly, young seedlings germinated from seeds treated with or without Ar/O_2_ were cut on small steam diagonally (45° from the horizontal) 0.4 cm above the germinated seeds two days after emergence. Scion (the portion removed) detached were grafted onto rootstock in four combinations (type 1: control self-grafting, type 2: Ar/O_2_ self-grafting, type 3: control rootstock + Ar/O_2_ scion, Type 4: Ar/O_2_ rootstock + control scion). Each graft was held together by a thin capillary tube placed over the graft. Grafted plants were then transferred to the hydroponic conditions. In following days, grafted plants showed adventitious rooting were discarded.

### Statistical analysis

Experiments were performed on the basis of randomized block design (CRBD) having three independent replications for each biological sample. Significance of each group data was analysed statistically at at P ≤ 0.05 by ANOVA one-way followed by Duncan’s Multiple Range Test (DMRT) in SPSS Statistics 20 software. The graphical figures were prepared using GraphPad Prism 6.

## Conclusion

This comprehensive study reveals that growth improvement in wheat due to LPDBD technique is mainly achieved through the tight regulation and signaling of H_2_O_2_ originated in root system but not due to the increased activity of the antioxidant enzyme in wheat plants. Findings of this study will be useful to optimize LPDBD technique in wheat or other crop plants with a view to improvement agronomic potentialities.

## Electronic supplementary material

Supplementary information
