# Supplementary material for: Mechanisms and Signaling Associated with LPDBD Plasma Mediated Growth Improvement in Wheat
Source: Sci Rep. 2018 Jul 12;8:10498. doi: 10.1038/s41598-018-28960-3 (PMC6043519; doi:10.1038/s41598-018-28960-3)
Supplement: Supplementary file 1 — Supplementary information [file 41598_2018_28960_MOESM1_ESM.pdf]

Mechanisms and Signaling Associated with LPDBD Plasma Mediated Growth Improvement in Wheat

Md Mosiur Rahman[1], Salek Ahmed Sajib[2], Md Sifat Rahi[2], Sharaban Tahura[1], Nepal Chandra Roy[3], Sarwar Parvez[1], Md Abu Reza[2], Mamunur Rashid Talukder[3], Ahmad Humayan Kabir[1*]

Supplementary Table S1. List of primers used in qPCR experiments.

Gene Name	Accession number	Primer sequences
TaActin	AY212324	Forward: GAATCCATGAGACCACCTAC<br>Reverse: AATCCAGACACTGTACTTCC
TaSOD	AF439787.2	Forward: CGAAGATTCCATTTCCCAGA<br>Reverse: TCGAGGATATCGGTGAAAGC
TaAPX	AB559521.1	Forward: TCCATCCAACCAAACCCGGAAA<br>Reverse: TGCCAATGTCCTTCTGTTCCCA
TaCAT	S81897.1	Forward: TTGGACCAGGCTTTATGGTC<br>Reverse: CCCTGTCACCACTCCAAGAT

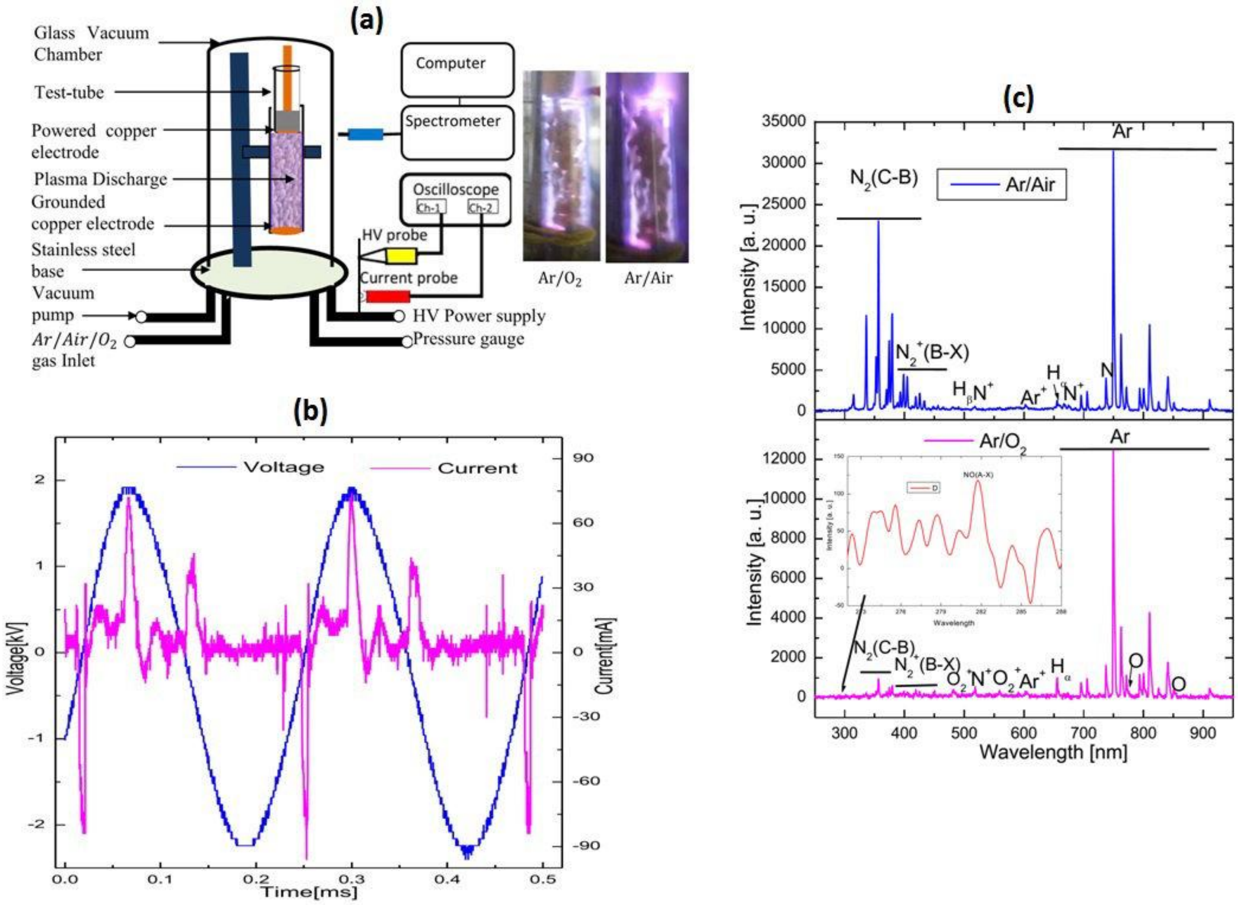

Supplementary Figure S1. Schematic diagram of (a) LPDBD plasma for wheat treatment with Ar/O_2 and Ar/Air gases, (b) V-I waveform of Ar/Air LPDBD plasma measured at applied voltage $5kV$ and electrode spacing 60 mm and (c) Emitted spectrum from Ar/O_2 and Ar/Air LPDBD plasmas at applied voltage $5kV$ and electrode spacing 60 mm.
